# Educational intervention to enhance the knowledge of Ghanaian health workers on Alzheimer’s disease and related dementias

**DOI:** 10.4102/phcfm.v14i1.3448

**Published:** 2022-04-26

**Authors:** Nana K. Ayisi-Boateng, Fred S. Sarfo, Douglas A. Opoku, Emmanuel K. Nakua, Emmanuel Konadu, Phyllis Tawiah, Ruth Owusu-Antwi, Akye Essuman, Bernard Barnie, Charles Mock, Peter Donkor

**Affiliations:** 1 Department of Medicine, School of Medicine and Dentistry, Kwame Nkrumah University of Science and Technology, Kumasi, Ghana; 2 Family Healthcare Services, Allen Clinic, Kumasi, Ghana; 3 Department of Epidemiology and Biostatistics, School of Public Health, Kwame Nkrumah University of Science and Technology, Kumasi, Ghana; 4 University Hospital, Kwame Nkrumah University of Science and Technology, Kumasi, Ghana; 5 Department of Psychiatry, Komfo Anokye Teaching Hospital, Kumasi, Ghana; 6 School of Medicine, University of Health and Allied Sciences, Ho, Ghana; 7 Department of Surgery, School of Medicine and Dentistry, Kwame Nkrumah University of Science and Technology, Kumasi, Ghana; 8 Department of Epidemiology, School of Public Health, University of Washington, Washington, United States of America

**Keywords:** Alzheimer’s, dementia, knowledge, health workers, Ghana

## Abstract

**Background:**

Alzheimer’s disease and related dementias (ADRDs) pose a major public health challenge in older adults. In sub-Saharan Africa, the burden of ADRD is projected to escalate amidst ill-equipped healthcare workers (HCWs).

**Aim:**

This study aimed to assess ADRD knowledge amongst Ghanaian HCWs and improve gaps identified through a workshop.

**Setting:**

Study was conducted among HCWs attending a workshop in Kumasi, Ghana.

**Methods:**

On 18 August 2021, a workshop on ADRD was organised in Kumasi, Ghana, which was attended by 49 HCWs comprising doctors, nurses, pharmacists, social workers and nutritionists. On arrival, they answered 30 pre-test questions using the Alzheimer’s Disease Knowledge Scale (ADKS). A post-test using the same questionnaire was conducted after participants had been exposed to a 4-h in-person educational content on ADRD delivered by facilitators from family medicine, neurology, geriatrics, psychiatry and public health.

**Results:**

The mean age of participants was 34.6 (± 6.82), mean years of practice was 7.7 (± 5.6) and 38.8% (*n* = 19) were nurses. The mean score of participants’ overall knowledge was 19.8 (± 4.3) at pre-test and 23.2 (± 4.0) at post-test. Participants’ pre-test and post-test scores improved in all ADKS domains. Factors associated with participants’ knowledge at baseline were profession, professional rank and the highest level of education attained. After adjusting for age and sex, participant’s rank, being a specialist (adjusted β = 14.44; 95% confidence interval [CI] = 7.03, 21.85; *p* < 0.001) was an independent predictor of knowledge on Alzheimer’s disease.

**Conclusion:**

Existing knowledge gaps in ADRD could be improved via continuous medical education interventions of HCWs to prepare healthcare systems in Africa for the predicted ADRD epidemic.

## Introduction

Alzheimer’s disease and related dementias (ADRDs) is typified as a progressive neurodegenerative disease characterised by cognitive decline, memory loss, impaired judgement and behavioural changes. Globally, about 43.8 million people live with dementia and Alzheimer’s disease is considered the commonest form.^1,2^ The prevalence of Alzheimer’s disease in older adults ≥ 65 years is reported to be from 2.4% to 15.8%.^1^ Alzheimer’s disease and related dementias has not been given much attention in developing countries, especially in sub-Saharan Africa. However, because of an ageing population, dementia is becoming more common in Africa with a reported prevalence of 1.0% – 5.3% and Alzheimer’s disease comprises 60.0% of dementia cases.^3^ Amongst Nigerians, an estimated 2.6% to 6.4% people are living with dementia^4,5^ and 13.6% of post-stroke survivors in Ghana have vascular dementia.^6^ It is projected that by 2040, developing countries will account for 71.0% of 81.1 million dementia cases.^3,7^

The regional differences in prevalence of ADRD may be due to different diagnostic criteria and screening tools, sociocultural dimensions such as stigma, which prevents early report of symptoms and various degrees of awareness or understanding about the disease.^8^ Changes in anatomy of neurons, establishment of adult brain structure, early life exposures and genetic factors have been postulated to underlie an individual’s risk of developing ADRD.^9^ However, its exact aetiology remains elusive, there is currently no definitive cure and knowledge about the disease is limited.

Undoubtedly, healthcare workers’ (HCWs’) knowledge of ADRD makes a significant difference in early detection, effective intervention, support for carers and progression of the disease. At least 25% of cases of ADRD with mild manifestations are missed at the primary care level.^1^ A study has predicted a 9.2 million decline in number of ADRD cases if an intervention is provided to achieve at least a one-year delay in disease onset and progression.^10^ However, there are knowledge gaps and a lack of awareness amongst HCWs about the disease and this has been documented elsewhere.^11^ The authors therefore sought to assess Ghanaian HCW’s knowledge on ADRD before and after participating in a workshop on the subject. They also solicited their resolutions and recommendations on measures to enhance knowledge and minimise the impact of the disease.

## Methods

### Study design

This was a pre- and post-test cross-sectional study involving a sample of healthcare professionals working in both public and private facilities in Kumasi, Ghana. Participants included doctors, nurses, pharmacists, social workers, nutritionists and public health practitioners working in primary, secondary and tertiary institutions who were invited to attend a workshop on ADRD on 18 August 2021.

### Workshop proceedings and data collection

As participants reported to the venue, they were welcomed and, after registration, a pre-test questionnaire using the Alzheimer’s Disease Knowledge Scale (ADKS) was shared with them. The ADKS is a 30 true-false questions validated tool, which has previously been used in other studies.^12,13^ Participants voluntarily provided answers to the questionnaire that were returned to workshop organisers before presentations by facilitators. The facilitators were a family physician, a neurologist, a geriatrician, a psychiatrist and a public health specialist. Topics treated ranged from epidemiology of the disease, clinical manifestations, diagnosis and treatment, providing support for families and carers and mental health issues associated with ADRD. After the presentations, a post-test using the same ADKS questionnaire was conducted to determine any change in participants’ knowledge.

Participants were placed in five heterogeneous groups of maximum ten members who listed personal resolutions on how they would apply the knowledge acquired during the workshop in their daily work and also provided a list of recommendations to policymakers on how to better address ADRD in Ghana.

### Statistical analysis

Demographic data were obtained from participants and the scores from the ADKS questionnaire. Data were entered into an excel spreadsheet for data quality management. Two independent data officers cleaned the data to ensure that there were no wrong and double entries. The data were merged and later checked for consistency. The data were exported to STATA version 16 for analysis. Mean and standard deviations were calculated. A *t*-test or one-way analysis of variance (ANOVA) test was conducted to assess the difference in health workers’ knowledge on Alzheimer’s disease using the ADKS at both pre-test and post-test. Univariate and multivariate linear regression analyses were performed to determine the independent predictors of health workers’ knowledge on Alzheimer’s disease. In the multivariate linear regression model (model 2), we adjusted for age and sex together with significant variables in the univariate model (model 1). In all analyses, *p*-values ≤ 0.05 were considered as significant at a 95% confidence interval (CI).

### Ethical considerations

Ethical approval was obtained from the Committee on Human Research Publications and Ethics at the School of Medicine and Dentistry, Kwame Nkrumah University of Science and Technology, Kumasi, Ghana (CHRPE/AP/122/21).

## Results

### Demographic characteristics of participants

The mean age of participants was 34.6 (± 6.8) years with a minimum age of 22 years and a maximum age of 50 years. More than half (51.0%, *n* = 25) of the participants were females. About 38.8% (*n* = 19) of the participants were nurses whilst over 24.5% of the participants were medical officers and pharmacists. The mean years of practice was 7.7 (± 5.6) with almost half (44.9%, *n* = 22) of the participants having practiced for between 1 and 5 years. Majority (59.2%, *n* = 29) of the participants were involved in the management of patients with Alzheimer’s disease (Table 1).

**TABLE 1:** Demographic characteristics of participants (*N* = 49).

Variable	Frequency (*n*)	Percentage	Range	Mean ± s.d.
**Age (years)**	-	-	22–50	34.60 ± 6.82
**Gender**	-	-	-	-
Male	24	49.0	-	-
Female	25	51.0	-	-
**Relationship status**				
Married	29	59.2	-	-
Divorced/separated	3	6.1	-	-
Single	17	34.7	-	-
**Highest level of education**				
Diploma	7	14.3	-	-
Graduate	25	51.0	-	-
Postgraduate	17	34.7	-	-
**Religion**				
Christian	47	95.9	-	-
Muslim	1	2.0	-	-
None	1	2.0	-	-
**Years of practice**	-	-	-	7.7 ± 5.6
1–5	22	44.9	-	-
6–10 years	12	24.5	-	-
11–15 years	11	22.4	-	-
> 15 years	4	8.2	-	-
**Place of practice**				
Primary/secondary level	23	46.9	-	-
Secondary/regional level	9	18.4	-	-
Tertiary level	17	34.7	-	-
**Profession**				
Doctor	16	32.7	-	-
Nursing staff	19	38.8	-	-
Pharmacist	7	14.3	-	-
Social worker	1	2.0	-	-
Others	6	12.2	-	-
**Rank**				
Specialist	9	18.4	-	-
Medical officer/pharmacist	12	24.5	-	-
PMO/PNO	2	4.1	-	-
SMO/SNO	5	10.2	-	-
NO/MO	7	14.3	-	-
SSM/SSN	4	8.2	-	-
SM/SN	2	4.1	-	-
Others	8	16.3	-	-

s.d., standard deviation; PMO/PNO, principal midwifery/nursing officer; SMO/SNO, senior midwifery/nursing officer; NO/MO, nursing officer/midwifery officer; SSM/SSN, senior staff midwife/senior staff nurse; SM/SN, staff midwife/staff nurse; Others, senior dietitian, research assistant, clinical psychologist.

### Participants’ knowledge on Alzheimer’s disease

With a maximum score of 30, the overall mean score of participants’ knowledge as measured by the ADKS was 19.8 (± 4.3) at pre-test and 23.2 (± 4.0) at post-test. There was an increase in the proportion of participants who had correct answers in all the seven domains of risk factors (6.12% vs 26.53%), symptoms (18.37% vs. 20.41%), course (26.53% vs 36.73%), assessment and diagnosis (38.78% vs 69.39%), treatment and management (57.14% vs. 65.31%) caregiving (2.04% vs. 36.73%) and life impact (42.86% vs. 67.35%).

Notably, participants with correct responses for several individual questions were low at baseline. The questions included: *When people with Alzheimer’s disease begin to have difficulty taking care of themselves, caregivers should take over right away* (40.82% of participants responded correctly). *It has been scientifically proven that mental exercise can prevent a person from getting Alzheimer’s disease* (38.78%) and *tremor or shaking of the hands or arms is a common symptom in people with Alzheimer’s disease* (44.90%). Post-test scores for these questions and majority of others improved (Table 2).

**TABLE 2:** Alzheimer’s Disease Knowledge Scale domains and proportion of participants with correct answers at pre-test and post-test.

Question	Correct answer	Pre-test		Post-test	
		*n*	%	*n*	%
**Life impact**					
People with Alzheimer’s disease are particularly prone to depression.	True	45	91.84	49	100.0
Most people with Alzheimer’s disease live in nursing homes.	False	35	71.43	40	81.63
It is safe for people with Alzheimer’s disease to drive, as long as they have a companion in the car at all times.	False	32	65.31	40	81.63
**Risk factors**					
It has been scientifically proven that mental exercise can prevent a person from getting Alzheimer’s disease.	False	19	38.78	24	48.98
People in their 30s can have Alzheimer’s disease.	True	25	51.02	24	48.98
Having high cholesterol may increase a person’s risk of developing Alzheimer’s disease.	True	32	65.31	41	83.67
Prescription drugs that prevent Alzheimer’s disease are available.	False	30	61.22	42	85.71
Having high blood pressure may increase a person’s risk of developing Alzheimer’s disease	True	34	69.39	46	93.88
Genes can only partially account for the development of Alzheimer’s disease.	True	35	71.43	37	75.51
**Symptoms**					
Tremor or shaking of the hands or arms is a common symptom in people with Alzheimer’s disease.	False	22	44.90	34	69.39
Trouble handling money or paying bills is a common early symptom of Alzheimer’s disease.	True	35	71.43	28	57.14
One symptom that can occur with Alzheimer’s disease is believing that other people are stealing one’s things.	True	38	77.55	48	97.96
Most people with Alzheimer’s disease remember recent events better than things that happened in the past.	False	28	57.14	30	61.22
**Assessment and diagnosis**					
If trouble with memory and confused thinking appears suddenly, it is likely because of Alzheimer’s disease	False	30	61.22	37	75.51
When a person with Alzheimer’s disease becomes agitated, a medical examination might reveal other health problems that caused the agitation.	True	43	87.76	47	95.92
Symptoms of severe depression can be mistaken for symptoms of Alzheimer’s disease.	True	42	85.71	46	93.88
Alzheimer’s disease is one type of dementia	True	46	87.76	49	100.0
**Treatment and management**					
People whose Alzheimer’s disease is not yet severe can benefit from psychotherapy for depression and anxiety	True	46	93.88	47	95.92
Poor nutrition can make the symptoms of Alzheimer’s disease worse	True	43	87.76	48	97.96
When a person has Alzheimer’s disease, using reminder notes is a crutch that can contribute to decline	False	35	71.43	35	71.43
Alzheimer’s disease cannot be cured.	True	43	87.76	46	93.88
**Care giving**					
People with Alzheimer’s disease do best with simple instructions giving one step at a time.	True	38	77.55	48	97.96
When people with Alzheimer’s disease begin to have difficulty taking care of themselves, caregivers should take over right away.	False	20	40.82	27	55.10
If a person with Alzheimer’s disease becomes alert and agitated at night, a good strategy is to try to make sure that the person gets plenty of physical activity during the day.	True	34	69.39	42	85.71
When people with Alzheimer’s disease repeat the same question or story several times, it is helpful to remind them that they are repeating themselves	True	25	51.02	34	69.39
Once people have Alzheimer’s disease, they are no longer capable of making informed decisions about their own care.	False	29	59.18	24	48.98
**Course**					
A person with Alzheimer’s disease becomes increasingly likely to fall down as the disease gets worse.	True	39	79.59	44	89.80
After symptoms of Alzheimer’s disease appear, the average life expectancy is 6 to 12 years	True	32	65.31	25	51.02
Eventually, a person with Alzheimer’s disease will need 24-h supervision	True	33	67.35	41	83.67
**Domain analysis** †					
Risk factors	-	3	6.12	13	26.53
Symptoms	-	9	18.37	10	20.41
Course	-	13	26.53	18	36.73
Assessment and diagnosis	-	19	38.78	34	69.39
Treatment and management	-	28	57.14	32	65.31
Caregiving	-	1	2.04	18	36.73
Life impact	-	21	42.86	33	67.35

† , Had all responses correct.

However, there were some individual questions for which the proportion of participants with correct responses dropped on the post-test. These were *people in their 30s can have Alzheimer’s disease* (51.02% vs 48.98%), *trouble in handling money or paying bills is a common early symptom of Alzheimer’s disease* (71.43% vs 57.14%), *once people have Alzheimer’s disease, they are no longer capable of making informed decisions about their own care* (59.18% vs 48.98%) and *after symptoms of Alzheimer’s disease appear, the average life expectancy is 6 to 12 years* (65.31% vs 51.02%) (Table 2).

### Relationship between demographic characteristics and participants’ knowledge on Alzheimer’s disease at pre-test and post-test

At pre-test and post-test, factors associated with participants’ knowledge were their profession (*p* = 0.007; *p* < 0.001), professional rank (*p* = 0.008; *p* < 0.001) and the highest level of education attained (*p* = 0.007; *p* = 0.008) (Table 3).

**TABLE 3:** Demographic characteristics and their relationship with Alzheimer’s knowledge at pre-test and post-test.

Variable	Pre-test	*p*	Post-test	*p*
	Mean ± s.d.		Mean ± s.d.	
**Age**	19.76 ± 4.28	0.493	23.16 ± 4.01	0.530
**Gender†**	-	0.473	-	0.641
Male	20.21 ± 4.49	-	23.48 ± 4.04	-
Female	19.32 ± 4.11	-	22.93 ± 4.04	-
**Profession**	-	0.007*	-	< 0.001*
Doctor	22.50 ± 3.71	-	25.92 ± 3.82	-
Nurse/midwife/healthcare assistant	17.84 ± 3.06	-	20.67 ± 2.79	-
Pharmacist	19.71 ± 4.92	-	24.43 ± 3.26	-
Social worker	13.00 ± 0.00	-	28.00 ± 0.00	-
Others	19.67 ± 4.97	-	25.75 ± 3.77	-
**Years of practice**	-	0.737	-	0.841
1–5 years	19.32 ± 4.11	-	23.62 ± 4.64	-
6–10 years	19.25 ± 4.33	-	23.07 ± 3.43	-
11–15 years	20.91 ± 5.01	-	23.00 ± 3.61	-
> 15 years	20.50 ± 3.79	-	21.80 ± 4.15	-
**Rank**	-	0.008*	-	< 0.001*
Specialist	23.44 ± 3.40	-	27.17 ± 2.86	-
Medical officer/pharmacist	21.67 ± 3.45	-	25.08 ± 3.43	-
PMO/PNO	19.00 ± 1.41	-	19.67 ± 0.58	-
SMO/SNO	18.80 ± 4.02	-	20.88 ± 2.30	-
NO/MO	16.71 ± 3.25	-	22.75 ± 1.50	-
SSM/SSN	18.50 ± 2.08	-	21.00 ± 4.24	-
SM/SN	15.00 ± 2.82	-	17.00 ± 2.00	-
Others	18.00 ± 5.24	-	24.71 ± 3.82	-
**Highest level of education**	-	0.007*	-	0.008*
Diploma	17.86 ± 2.41	-	20.38 ± 3.42	-
Graduate	18.56 ± 4.08	-	22.64 ± 3.84	-
Postgraduate	22.29 ± 4.13	-	25.38 ± 3.54	-
**Involvement in Alzheimer’s care**	-	0.995	-	0.685
Yes	19.76 ± 4.45	-	22.91 ± 3.81	-
No	19.75 ± 4.14	-	23.38 ± 4.23	-
**Level of practice**	-	-	-	0.368
Primary level	15.00 ± 4.24	-	20.40 ± 3.91	-
District level	19.23 ± 3.88	-	23.19 ± 3.75	-
Secondary/regional level	18.44 ± 4.07	-	23.11 ± 4.59	-
Tertiary level	21.65 ± 4.33	-	24.14 ± 4.00	-

s.d., standard deviation; PMO/PNO, principal midwifery/nursing officer; SMO/SNO, senior midwifery/nursing officer; NO/MO, nursing officer/midwifery officer; SSM/SSN, senior staff midwife/ senior staff nurse; SM/SN, staff midwife/staff nurse; Others, senior dietician, research assistant, clinical psychologist.

* , *p* < 0.05.

† , Analysed using t-test.

### Predictors of participants’ knowledge on Alzheimer’s disease at both pre-test and post-test

The results of the multiple linear regression after adjusting for age and sex showed that participant’s rank was an independent predictor of knowledge on Alzheimer’s disease using ADKS at pre-test. Specialist (adjusted [adj] β = 14.44; 95% CI = 7.03, 21.85; *p* < 0.001) and medical officer/pharmacist (adj β = 13.99; 95% CI = 6.87, 21.10; *p* < 0.001) were the most significant predictors of participant’s knowledge. This model explained about 40.9% variance of participant’s knowledge on Alzheimer’s disease using ADKS at pre-test (Table 4).

**TABLE 4:** Linear regression model predicting the overall knowledge of Alzheimer’s disease at pre-test.

Predictors	Model 1					Model 2				
	Unadjusted *β*	CI	s.e.	*t*	*p*	Adjusted *β*	CI	s.e.	*t*	*p*
**Profession**										
Doctor	4.62	2.04 to 7.27	1.3	3.59	0.001 *	−5.28	−12.57 to 2.00	3.58	−1.48	0.149
Nursing staff	1.00	-	-	-	-	1.00	-	-	-	-
Pharmacist	1.87	−1.54 to 5.28	1.69	1.11	0.274	−1.98	−10.10 to 6.14	3.99	−0.5	0.623
Social worker	−4.84	−12.75 to 3.06	3.92	−1.23	0.224	4.83	−9.13 to 18.80	6.86	0.7	0.486
Others	1.83	−1.78 to 5.43	1.79	1.02	0.314	5.68	−2.93 to 14.30	4.23	1.34	0.189
**Years of practice**										
1–5 years	1.00	-	-	-	-	-	-	-	-	-
6–10 years	−0.07	−3.22 to 3.08	1.56	−0.04	0.965	-	-	-	-	-
10–15 years	1.59	−1.65 to 4.83	1.61	0.99	0.328	-	-	-	-	-
> 15 years	1.18	−3.59 to 5.95	2.37	0.5	0.62	-	-	-	-	-
**Rank**										
Specialist	5.44	1.80 to 9.09	1.8	3.02	0.004*	14.44	7.03 to 21.85	3.64	3.97	< 0.001*
Medical officer/pharmacist	3.67	0.24 to 7.09	1.7	2.16	0.037*	13.99	6.87 to 21.10	3.49	4	< 0.001*
PMO/PNO	1.00	−4.93 to 6.93	2.94	0.34	0.735	9.61	0.86 to 18.37	4.3	2.24	0.032*
SMO/SNO	0.8	−3.48 to 5.08	2.12	0.38	0.708	11.39	2.13 to 20.64	4.54	2.51	0.017*
NO/MO	−1.29	−5.17 to 2.60	1.92	−0.67	0.508	7.26	−2.04 to 16.57	4.57	1.59	0.122
SSM/SSN	0.50	−4.10 to 5.10	2.28	0.22	0.827	3.62	−3.59 to 10.83	3.54	1.02	0.314
SM/SN	−3.00	−8.93 to 2.93	2.94	−1.02	0.313	0.77	−8.69 to 10.23	4.64	0.17	0.869
Others	1.00	-	-	-	-	1.00	-	-	-	-
**Highest level of education**										
Diploma	1.00	-	-	-	-	1	-	-	-	-
Graduate	0.70	−2.68 to 4.08	1.68	0.42	0.677	−4.17	−12.34 to 4.00	4.01	−1.04	0.307
Postgraduate	4.44	−2.68 to 4.08	1.76	2.52	0.015*	1.09	−6.75 to 8.93	3.85	0.28	0.778

Note: P-values in bold is statistically significant. Adjusted for age and sex together with significant variables in Model 2. Model 2 – Adjusted β: Adjusted *R*^2^ = 0.409; Prob. > *F* = 0.00.

CI, confidence interval; s.e., standard error; PMO/PNO, principal midwifery/nursing officer; SMO/SNO, senior midwifery/nursing officer; NO/MO, nursing officer/midwifery officer; SSM/SSN, senior staff midwife/ senior staff nurse; SM/SN, staff midwife/staff nurse; Others, senior dietician, research assistant, clinical psychologist.

* , *p* < 0.05.

At post-test, in model 2, after adjusting for age and sex, the results showed that participant’s rank was independently associated with knowledge on Alzheimer’s disease using ADKS. Specialist (adj β = 7.04; 95% CI = 0.40, 13.68; *p* = 0.039) was independently associated with high knowledge score. This model explained about 42.9% variance of participant’s knowledge on Alzheimer’s disease using ADKS at post-test (Table 5).

**TABLE 5:** Regression model predicting the overall knowledge of Alzheimer’s disease at post-test.

Predictors	Model 1					Model 2				
	Unadjusted *β*	CI	s.e.	*T*	*p*	Adjusted *β*	CI	s.e.	*t*	*p*
**Profession**										
Doctor	5.26	3.01 to 7.50	1.11	4.72	< 0.001*	0.11	−6.33 to 6.55	3.16	0.03	0.973
Nursing staff	1.00	-	-	-	-	1.00	-	-	-	-
Pharmacist	3.76	0.96 to 6.56	1.39	2.71	0.010*	0.57	−6.06 to 7.20	3.25	0.18	0.862
Social worker	7.33	0.68 to 13.99	3.30	2.22	0.032*	8.11	−1.07 to 17.29	4.50	1.80	0.081
Others	5.08	1.56 to 8.61	1.75	2.91	0.006*	6.10	−1.03 to 13.23	3.50	1.75	0.091
**Years of practice**										
1–5	1.00	-	-	-	-	-	-	-	-	-
6–10	−0.55	−3.40 to 2.30	1.41	−0.39	0.701	-	-	-	-	-
11–15	−0.62	−3.91 to 2.67	1.63	−0.38	0.707	-	-	-	-	-
> 15	−1.82	−5.93 to 2.29	2.04	−0.89	0.377	-	-	-	-	-
**Rank**										
Specialist	2.45	−1.03 to 5.93	1.72	1.42	0.162	7.04	0.40 to 13.68	3.26	2.16	0.039*
Medical officer/pharmacist	0.36	−2.57 to 3.30	1.45	0.25	0.804	5.86	−0.53 to 12.24	3.13	1.87	0.071
PMO/PNO	−5.05	9.36 to −0.73	2.14	−2.36	0.023*	−1.16	−7.83 to 5.51	3.27	−0.35	0.726
SMO/SNO	−3.84	−7.08 to −0.60	1.60	−2.39	0.021*	0.99	−5.53 to 7.51	3.20	0.31	0.759
NO/MO	−1.96	−5.89 to 1.96	1.94	−1.01	0.318	3.27	−4.13 to 10.66	3.63	0.90	0.375
SSM/SSN	−3.71	−7.38 to −0.05	1.81	−2.05	0.047*	−0.13	−6.40 to 6.14	3.07	−0.04	0.967
SM/SN	−7.71	−12.03 to −3.40	2.14	−3.61	0.001*	−3.96	−10.60 to 2.69	3.26	−1.21	0.234
Others	1.00	-	-	-	-	1.00	-	-	-	-
**Highest level of education**										
Diploma	1.00	-	-	-	-	1.00	-	-	-	-
Graduate	2.27	−0.75 to 5.28	1.50	1.51	0.137	−1.98	−6.30 to 2.34	2.12	−0.94	0.356
Postgraduate	5.00	1.79 to 8.21	1.59	3.14	0.003*	−1.38	−6.22 to 3.46	2.37	−0.58	0.566

Note: Adjusted for age and sex together with significant variables in Model 2. Model 2 – Adjusted β: Adjusted *R*^2^ = 0.429; Prob > *F* = 0.002.

CI, confidence interval; s.e., standard error; PMO/PNO, principal midwifery/nursing officer; SMO/SNO, senior midwifery/nursing officer; NO/MO, nursing officer/midwifery officer; SSM/SSN, senior staff midwife/ senior staff nurse; SM/SN, staff midwife/staff nurse; Others, senior dietitian, research assistant, clinical psychologist.

* , *p* < 0.05.

### Participants’ resolutions and recommendations

From the five groups with interprofessional representation, some resolutions and recommendations made included the need for greater multidisciplinary and multisectorial collaboration, increased public education on ADRD, provision of financial and social support and expansion of geriatric services to cater for people living with ADRD and their families (Table 6).

**TABLE 6:** Participants’ resolutions and recommendations.

No.	Resolutions and recommendations
1	There is the need to take detailed and accurate patient history.
2	There is the need for collaboration between general practitioners, the mental health units and community health nurses.
3	Patients should be involved in their diet planning.
4	There should be increased public education on the condition.
5	Social workers should be involved in the care of patients.
6	Stigmatisation and profiling of patients should be discouraged.
7	Toxicity and efficacy of medications should be monitored.
8	Counselling and emotional support should be provided for caregivers of people with dementia.
9	Clinical trials on Alzheimer’s disease should be explored.
10	Encourage physical activities in management of Alzheimer’s disease.
11	Include Alzheimer’s disease medications on National Health Insurance Scheme (NHIS).
12	Adopt multidisciplinary and holistic approach in the management of Alzheimer’s disease.
13	Geriatric, family medicine and other related specialist clinics should be established at every hospital.
14	Hospital environment should be friendly to welcome patients with ADRD.
15	There should be a national policy for financial support for patients and families with persons suffering from Alzheimer’s disease.
16	Review patient’s drugs in order to stop non-essential medications.
17	Avoid unnecessary physical restraints on patients and protecting patients from potential physical harm.
18	Form dementia support group for patients with ADRD and their caregivers.
19	Provision of a toll-free hotline for caregivers of ADRD patients.
20	More training for health workers and caregivers on Alzheimer’s disease.

ADRD, Alzheimer’s disease and related dementias.

## Discussion

By 2050, the number of people living with ADRD is projected to hit 106.8 million^10^ and this calls for increased awareness, identification of knowledge gaps and institution of appropriate educational interventions for relevant stakeholders. Comparable with our findings, an earlier study found the ADKS questionnaire to be a useful tool in identifying education needs of health workers and assessing effectiveness of education efforts.^12^ In this study, in which 59.2% of the participants indicated some involvement in managing patients with Alzheimer’s disease, their mean ADKS at pre-test was 19.8 (± 4.28) and this significantly increased to 23.2 (± 4.01). These mean scores amongst HCWs were higher than 11.7 ± 3.02 obtained amongst elderly Egyptians^14^ but lower than 20.15 to 27.40 reported by Carpenter et al in the United States.^13^ Our participants’ knowledge scores increased in all seven ADKS domains. In a previous study, domains with significant ADKS scores were assessment, treatment and management and the participants recorded the least scores in the risk factors and prevention domains.^13^

The ADKS is predicted to produce different scores for various categories of health workers, based on their experience and theoretical knowledge.^12^ In our study, type of profession and years of practice did not impact the ADKS scores. However, a participant’s rank was an independent predictor of pre- and post-test scores. Those with post-graduate medical qualification, considered specialists and comprised 18.4% of the participants, recorded the highest overall scores. These were specialists in family medicine, internal medicine and psychiatry who practice at primary, secondary and tertiary level facilities. This implies that in our setting, specialists can lead capacity-building efforts across various levels of care. It also suggests the need to intensify education interventions for the 81.6% HCWs below the specialist rank. The bulk of these HCWs work in primary care settings. Perhaps, the 4-h duration of the workshop was inadequate and this category of HCWs may benefit from a more prolonged training engagement.

The baseline (pre-test) scores and the improvement in scores in both content areas and in specific questions point towards areas that might need extra attention in future efforts to build knowledge on ADRD in the HCW in Ghana and other similar countries.

Resolutions shared by the participants are quite insightful and critical to addressing ADRD challenges, which may be provider-related, patient-related or health system-related. Participants recommended inclusion of medications for managing ADRD in the National Health Insurance Scheme (NHIS) and provision of financial support for families living with the disease. With an estimated $156 billion worldwide direct cost of dementia and a projection of higher costs in developing countries,^8^ there is the need for system-based financial cushioning. In spite of its challenges, Ghana’s NHIS and health insurance schemes in other parts of the world have been a source of financial reprieve for the poor and vulnerable.^15,16^ Another recommendation by participants is the provision of geriatric services at various health institutions in Ghana and creation of an aged-friendly and supportive environment. This calls for intense education. An interprofessional, team-based approach in which various categories of health workers are trained and their expertise harnessed was an important recommendation by our participants and this has been touted as an innovative strategy.^17,18^ Our workshop, which assembled a wide spectrum of health workers, has therefore provided a foundation for future collaborations in ADRD-related activities in Ghana.

## Strengths and limitations

The findings of this study are very relevant, as to our knowledge, this is the first to be carried out in sub-Saharan Africa using ADKS among HCWs whose role in addressing the looming ADRD epidemic cannot be overemphasised. The sociodemographic and professional characteristics of the participants were varied and quite representative of HCWs in Ghana. Previous studies have been undertaken in Egypt and the United States amongst different populations. Unlike these earlier studies, ours goes beyond highlighting domains with knowledge gaps to analysing pre- and post-test scores after a training session. Our study is limited by the relatively small sample size and the 4-h engagement with participants. In addition, being a cross-sectional study, we are limited in our ability to generalise our findings. Future studies should target a larger population of participants.

## Conclusion

Knowledge gaps on ADRD exist amongst Ghanaian HCWs and educational intervention where local expertise help train a sample of HCWs improved scores on ADRD by four units. Amongst others, we have recommended NHIS funding of ADRD management, intensification of education at the primary care level, expansion of geriatric services and interprofessional collaboration. Increased awareness amongst the general population and training of a critical mass of health professionals are needed to care for sufferers and support families grappling with the debilitating effects of ADRD.
